# Calmodulin-Connexin Partnership in Gap Junction Channel Regulation-Calmodulin-Cork Gating Model

**DOI:** 10.3390/ijms222313055

**Published:** 2021-12-02

**Authors:** Camillo Peracchia, Lillian Mae Leverone Peracchia

**Affiliations:** Department of Pharmacology and Physiology, School of Medicine and Dentistry, University of Rochester, 601 Elmwood Avenue, Rochester, NY 14642, USA; lmperacchia@gmail.com

**Keywords:** gap junctions, connexins, innexins, channel gating, calcium, calmodulin, cell communication, cell-to-cell channels, cell coupling, cell uncoupling

## Abstract

In the past four decades numerous findings have indicated that gap junction channel gating is mediated by intracellular calcium concentrations ([Ca^2+^_i_]) in the high nanomolar range via calmodulin (CaM). We have proposed a CaM-based gating model based on evidence for a direct CaM role in gating. This model is based on the following: CaM inhibitors and the inhibition of CaM expression to prevent chemical gating. A CaM mutant with higher Ca^2+^ sensitivity greatly increases gating sensitivity. CaM co-localizes with connexins. Connexins have high-affinity CaM-binding sites. Connexin mutants paired to wild type connexins have a higher gating sensitivity, which is eliminated by the inhibition of CaM expression. Repeated trans-junctional voltage (Vj) pulses progressively close channels by the chemical/slow gate (CaM’s N-lobe). At the single channel level, the gate closes and opens slowly with on-off fluctuations. Internally perfused crayfish axons lose gating competency but recover it by the addition of Ca-CaM to the internal perfusion solution. X-ray diffraction data demonstrate that isolated gap junctions are gated at the cytoplasmic end by a particle of the size of a CaM lobe. We have proposed two types of CaM-driven gating: “Ca-CaM-Cork” and “CaM-Cork”. In the first, the gating involves Ca^2+^-induced CaM activation. In the second, the gating occurs without a [Ca^2+^]_i_ rise.

## 1. Introduction

Direct ionic communication between electrically excitable cells was discovered in the early 20th century [1,2], but for many decades this form of cell–cell communication was thought to be a property of excitable cells only. Therefore, everyone was surprised when in the 1950s direct cell-to-cell communication was found to exist in the cells of virtually all tissue [3,4,5,6]. In the mid-1960s, convincing evidence that this form of cell coupling could be regulated down to complete electrical and metabolic cell–cell uncoupling was reported [3,7,8,9]. However, some hints of the existence of cell–cell uncoupling mechanisms originated almost a century earlier. Indeed, in 1877 Engelmann reported that damaged cardiac cells became independent from their neighboring cells as they died [10]. This phenomenon, called “healing over”, is now known as “cell-to-cell uncoupling”, a property of all coupled cells, mediated by the chemical gating mechanism of gap junction channels; rev. in [11,12,13,14,15].

## 2. Cytosolic Calcium and Gap Junction Channel Gating

Nearly a century after Engelmann’s discovery [10], Jean Délèze reported that in cut cardiac fibers “healing over” only occurred in the presence of external Ca^2+^ [16], suggesting that the rise in intracellular calcium concentration ([Ca^2+^_i_]) caused by Ca^2+^ influx played a role in the regulation of gap-junctional communication. Soon after, the Ca^2+^-role in cell uncoupling was confirmed by evidence that cell–cell communication was lost in cells subjected to treatments that increase the [Ca^2+^]_i_ [9,15,17,18,19,20,21,22,23,24,25].

The [Ca^2+^]_i_ effect on gating has been questioned for more than four decades. Early studies suggested that a [Ca^2+^]_i_ as high as 40–400 µM is needed [26,27]. In contrast, numerous more recent reports indicate that a much lower [Ca^2+^]_i_, ranging from ~100 nM to low µM is effective. Data for the effectiveness of nanomolar [Ca^2+^]_i_ were first reported in a study [28,29] in which [Ca^2+^]_i_ of 251 nM (at pH 7.4), ~400 nM (at pH 7.0) and 2.5 µM (at pH = 6.5) were proven effective in uncoupling cardiac cell pairs, one of which was mechanically perforated to allow the influx of extracellular solutions well buffered for Ca^2+^ and H^+^. More recently, a treatment with ionomycin and gramicidin of arterially perfused rabbit papillary muscle uncoupled the cells at ~685 nM or greater [Ca^2+^]_i_ [30]; these data were confirmed in cells subjected to ischemia/reperfusion [30]. Low [Ca^2+^]_i_ proved effective on gating in many other cells, such as crayfish giant axons [31,32], rat lacrimal cells [33], Novikoff hepatoma cells [34,35], astrocytes [36,37,38], lens cultured cells [39], pancreatic β-cells [40], human fibroblasts [41], and Cx43-expressing cultured cells [42]. Mammalian pancreatic and lacrimal gland cells briefly uncoupled when secretion was stimulated by the application of acetylcholine or other secretagogues at concentrations below those required for maximal secretion [43,44,45], as well as by depolarization or cyclic nucleotide load [46,47]. In pancreatic acinar cells, even the treatment with secretagogues at concentrations capable of stimulating maximal secretory activity increases [Ca^2+^]_i_ only from 180 nM to 860 nM [48], further supporting the effectiveness on channel gating of nanomolar [Ca^2+^]_i_.

We have studied, with Ca^2+^- and pH-sensitive microelectrodes, the relationship between junctional electrical resistance (Rj), [Ca^2+^]_i_ and [H^+^]_i_ in crayfish septate axons uncoupled by low pH_i_ [32]. Cytosolic acidification (pH_i_ = 6.3), caused by the application of Na^+^-acetate, increased [Ca^2+^]_i_ by approximately one order of magnitude, from resting values of 100–300 nM, and greatly increased Rj, indicating that that crayfish gap junctions are sensitive to low µM [Ca^2+^]_i_ [32]. The time course of Rj and [Ca^2+^]_i_ matched well, while that of Rj and [H^+^]_i_ did not [32].

In order to more accurately determine the [Ca^2+^]_i_ effective on gating, we studied Novikoff hepatoma cell pairs by double whole-cell clamp [34,35]. In these Cx43-expressing cells, Ca^2+^_i_-gating sensitivity was tested by monitoring the decay of junctional conductance (Gj) at different [Ca^2+^]_i_ (buffered with BAPA), at pH_i_ = 7.2 or 6.1 (buffered with HEPES and MES, respectively). Channel gating was activated by [Ca^2+^]_i_ ranging from 500 nM to 1 µM, irrespective of pH_i_ [34]. With [Ca^2+^]_i_ = 0.5–1.0 µM, the Gj dropped to ~25% of the initial values with mean τ’s of 5.9 and 6.2 min, at pH_i_ = 6.1 and 7.2, respectively. With [Ca^2+^]_i_ = 3 µM, the cells uncoupled in <1 min (τ = ~20 s) [34]. The effectiveness of high nanomolar [Ca^2+^]_i_ on gating was confirmed in the same cells with brief (20 s) exposures to 20 µM arachidonic acid [35].

Similarly, a [Ca^2+^]_I_ < 0.5–1 µM blocked the cell–cell diffusion of Lucifer Yellow in chicken-lens-cultured cells [39], and nanomolar [Ca^2+^]_i_ drastically reduced the Gj in pancreatic β-cells, with a temperature drop from 37° to 30° and an external [Ca^2+^]_o_ rise from 2.56 mM to 7.56 mM [40]. Gating sensitivity to nM [Ca^2+^]_i_ was also reported in astrocytes injected with Lucifer Yellow and Ca^2+^ [36]. In these cells, nanomolar [Ca^2+^]_i_ prevented cell–cell dye transfer independently of pH_i_; the dye transfer was blocked by [Ca^2+^]_i_ ranging from 150–600 nM [36]. Consistent with these findings is a report that the addition of 20 mM of BAPTA to the patch pipette solutions substantially improves coupling between astrocytes [37], which indicates that gating may even be sensitive to basal [Ca^2+^]_i_. Dye coupling was also blocked in cultured astrocytes treated with ionomycin, which increased the [Ca^2+^]_i_ to 500 nM [38], and similar values were reported in lens-cultured cells [49]. In murine Neuro-2a cells (N2a) expressing Cx43, ionomycin treatment increased the Ca^2+^-influx and reduced the Gj by 95% [42], as the [Ca^2+^]_i_ increased from ~80 to ~250 nM. All of these data confirm the idea that Ca^2+^_i_ is a fine modulator of gap-junctional coupling.

## 3. Evidence for Calmodulin Role in Gap Junction Channel Gating

As gap junction proteins do not have highly sensitive intracellular Ca^2+^-binding sites, the data described in the previous chapter strongly suggest that Ca^2+^_i_ affects gating via an intermediate component. Indeed, since the early eighties we have proposed calmodulin (CaM) as the intermediate of the Ca^2+^ gating effect; rev. in: [50,51,52].

In 1981, Johnston and Ramón reported that crayfish giant axons lose their cell–cell gating sensitivity to increased Ca^2+^_i_ and/or decreased pH_i_ when they are internally perfused [53]. Their data, confirmed by Arellano and coworkers [54], induced them to suggest that a soluble intermediate mediates the Ca^2+^/H^+^-induced cell uncoupling [53]. In the same year, we first suggested CaM as the soluble intermediate of Ca^2+^_i_-induced gating [55,56]. Our idea was also supported by evidence for CaM binding to the gap junction protein connexin32 (Cx32) and to gap junction fragments from crayfish hepatopancreas [57,58].

**Figure 1:**
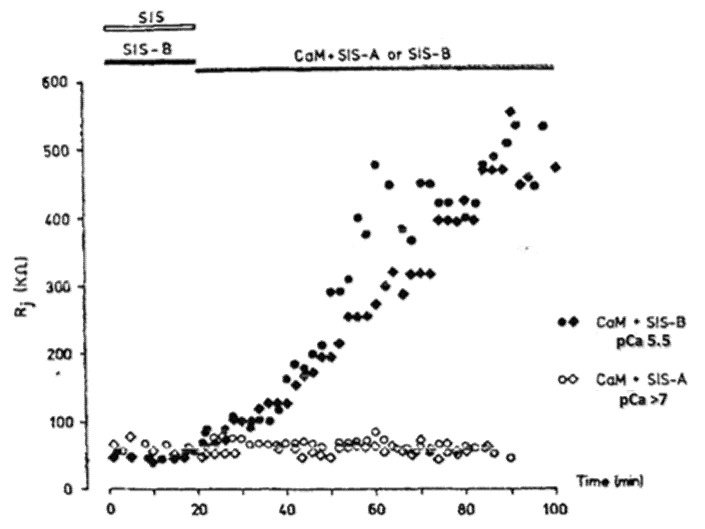
Changes in Junctional Resistance (Rj) in crayfish lateral giant axons in which one axon of the coupled pair was internally perfused with either of the following Standard Internal solutions (SIS): SIS (no added Ca^2+^, 0.1 mM EGTA, pH 7.1); SIS-A (no added Ca^2+^, 10 mM EGTA, pH 7.1, pCa > 7); SIS-B (1 mM CaCl_2_, 0.1 mM EGTA, pH 7.1, pCa 5.5); CaM + SIS-A or SIS-B (10 μM CaM, pH 7.1). Rj does not increase in the absence of CaM, either in the absence of Ca^2+^ (SIS) or with 1 mM Ca^2+^ (SIS-B), but does so greatly in the presence of Ca^2+^ + 10 μM CaM (CaM+SIS-B). In the authors’ words: “All data points were included in this figure, since the trend illustrated was observed in five other experiments with prolonged perfusion of calmodulin and high calcium”. Reproduced with permission from ref. [54].

**Figure 2:**
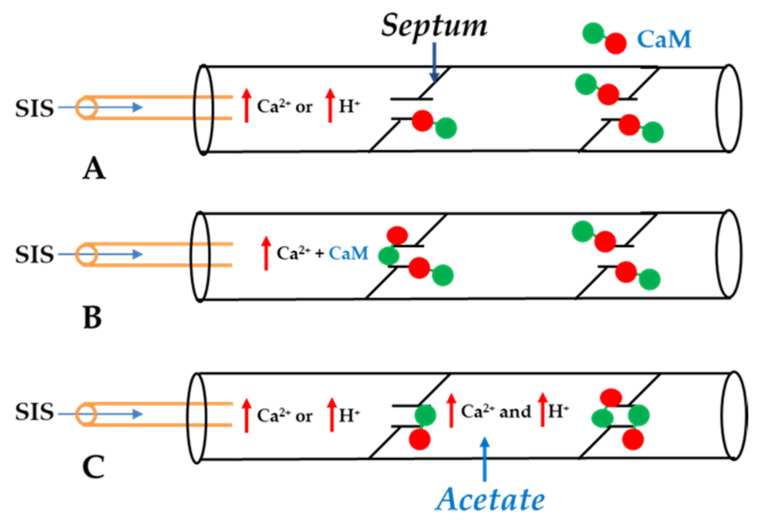
Summary of data from refs. [53,54,59]. While internal perfusion of crayfish lateral giant axons with Standard Internal Solution (SIS) with high [Ca^2+^] and/or [H^+^] does not induce channel gating (**A**), addition of 10 μM CaM to internal solutions with high [Ca^2+^] does (**B**). Axons internally perfused with high [Ca^2+^] and/or high [H^+^] without CaM uncouple with extracellular perfusion of Standard Extracellular Solution (SES) containing 205 mM Na-acetate (**C**), as acetate increases [Ca^2+^]_i_ by increasing [H^+^]_i_ in the un-perfused axon segment. (Red circle: CaM’s C-lobe; Green circle: CaM’s N-lobe).

In 1988, Arellano and coworkers provided convincing evidence that CaM is in fact the soluble intermediate that had been washed out by the internal perfusion of crayfish axons [54] because when the lateral giant axons were internally perfused with Ca-CaM (pCa 5.5; CaM + SIS-B), the Rj increased from the control values of ~60 kΩ to 500–600 kΩ in ~60 min (Figure 1). In contrast, the axons perfused either with CaM in low Ca^2+^ solutions (pCa > 7; CaM + SIS-A), with CaM-free high Ca^2+^ solutions (pCa 5.5; SIS-B) or with Ca-free solutions (SIS), maintained the Rj at control levels during the 60 min perfusion time (Figure 1). Figure 2 schematically summarizes the results of Arellano and coworkers [54,59]. The same results were reported with either only one axonal segment perfused (Figure 2) or with both segments perfused. Significantly, while 20 min of the internal perfusion of one axon segment with 1 mM Ca^2+^ in the absence of CaM did not change the Rj, the subsequent addition of Na^+^-acetate to the external solution, while maintaining the same internal solution, increased the Rj to ~400 kΩ [54]. This is very significant for two reasons: first, it proves that the septum was not damaged by the SIS perfusion; second, it proves that the uncoupling effect of acetate on the intact axon segment is not just due to an increase in [H^+^]_i_ [59], but rather to an acetate-induced rise in [Ca^2+^]_i_ resulting from the drop in pH_i_ (Figure 2), as also reported by us with different methods [32] (see in the previous).

**Figure 3:**
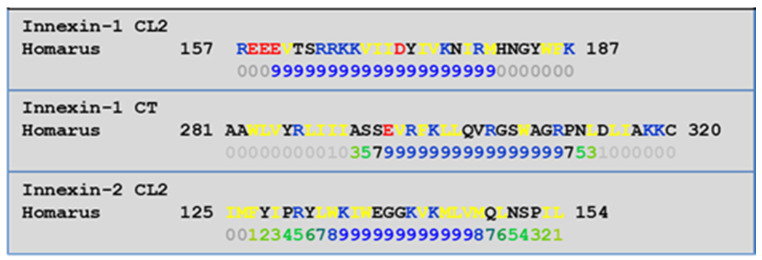
Innexins’ CaM-binding sites at CL2 and CT domain.

Crayfish express innexins rather that connexins, but innexins are very similar to connexins and contain CaM-binding sites. In crayfish giant axons both innexin-1 and innexin-2 are expressed [60]. Innexin-1 and -2 contain CaM-binding sites at the CT and CL2 domains (Figure 3). The CaM-binding prediction to the CT and CL2 domains of these innexins were identified by means of a computer program developed at the University of Toronto (http://calcium.uhnres.utoronto.ca/ctdb/ctdb/sequence.html, accessed on 28 November 2021. Copyright © 2021 Ikura Lab, Ontario Cancer Institute. All Rights Reserved).

In 1986, Arellano and coworkers also confirmed their earlier data of gating insensitivity to H^+^_i_ [59]. In this study, a glass capillary was inserted into one of the axons and one side of the junction was perfused with solutions of pH 7 or 6 (Figure 2A), while monitoring the Rj. Significantly, the Rj remained unchanged when the pH of the perfusate was lowered from 7.1 to 6.0 [59]. We have confirmed the absence of a direct effect of low pH_i_ on gating in crayfish axons [32], *Xenopus* oocyte pairs [61], and Novikoff hepatoma cell pairs [34]. In the Novikoff cells, we monitored the Gj at different pCa (9, 6.9, 6.3, 6, and 5.5; buffered with BAPTA) and pH_i_ (7.2 or 6.1; buffered with HEPES an MES, respectively). No significant difference in the Gj was observed between pH_i_ 7.2 and 6.1 as long as the [Ca^2+^]_i_ was carefully buffered with BAPTA [34].

**Figure 4:**
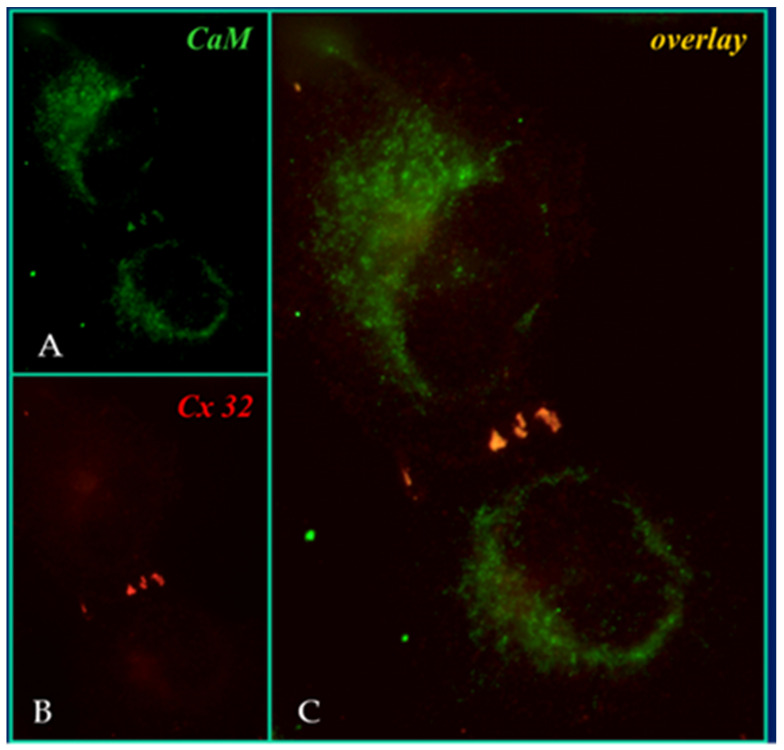
Immuno-fluorescence labeling of CaM (**A**) and Cx32 (**B**) in HeLa cells. The overlay of (**A**,**B**) is shown in (**C**). Cx32 and CaM colocalize at three punctuated areas of cell–cell contact. From ref. [72].

In the four decades that followed our reports of the early eighties [55,56,62], CaM participation in channel gating has been confirmed by multiple data generated by a variety of experimental procedures that include: treatment with CaM blockers [42,49,55,56,62,63,64,65,66,67], inhibition of CaM expression [68,69,70], overexpression of a CaM mutant (CaMCC) with higher Ca^2+^ sensitivity [71,72], colocalization of CaM and gap junctions by immune-fluorescence microscopy [71,72] (Figure 4), intracellular perfusion of crayfish axons with CaM-containing solutions [54], and in vitro testing of CaM binding to connexins [57,58,71,72] and synthetic connexin peptides mimicking CaM-binding sites of various connexins [42,52,73,74,75,76,77,78,79,80]; rev. in: [50,51,52].

After our early evidence for a CaM role in gap junction channel function [55,62,63,81], numerous other membrane channels have been found to directly involve CaM in their gating mechanisms. Indeed, there are an increasing number of channels regulated by CaM. In addition to connexins, they include: voltage-gated calcium (VGCC, CaV) channels, sodium (VGSC, NaV) channels, potassium channels (VGPC, KV), small conductance calcium-activated K^+^ channels (SK), inwardly rectifying potassium channels (Kir, IRK), cyclic nucleotide-gated channels (CNG), ryanodine receptors (RyR), and transient receptor potential channels (TRP), rev. in: [82,83], as well as the water channel aquaporin-0 AQP0), also known as the eye lens protein MIP26 [84,85,86,87,88,89,90].

CaM is an acidic protein of 148 amino acids, whose sequence is very well preserved from plants to mammals. It is a dumbbell shaped protein, ~65 Å long, made of two fairly spherical lobes of ~35 × 25 Å in size, called the N-lobe and the C-lobe [91]. A short NH_2_-terminus is followed by the N-lobe, which is linked to the C-lobe by a flexible amino acid chain. Each of the two lobes has two domains, known as EF-hands [92], which bind Ca^2+^ with nanomolar affinity. The Ca^2+^ affinity of the C-lobe is greater than that of the N-lobe by approximately one order of magnitude. Ca^2+^-binding to Ca^2+^-free CaM (apo-CaM) induces conformational changes that unmask a hydrophobic pocket in each lobe. Ca^2+^-CaM (holo-CaM) interacts with a receptor domain, usually made of a basic amphiphilic alpha-helix, by binding to it hydrophobically and electrostatically.

## 4. Calmodulin-Connexin Interaction—Are There Calmodulin Binding Sites in Connexins?

Hertzberg and Gilula first reported the CaM binding to Cx32 in gel overlays [57]. Their data were soon confirmed by several studies [58,76,93]. The CaM-Cx32 interaction was also indirectly supported by evidence that CaM prevents Cx32 proteolysis by m-calpain [94] and Cx32 phosphorylation by the EGF receptor tyrosine kinase [95]. CaM binding to connexins is also suggested by in vitro data demonstrating CaM participation in the oligomerization of Cx32 into connexons [96].

### 4.1. NH_2_-Terminus (NT) and Initial COOH-Terminus (CT1) CaM-Binding Sites

In 1988, we first identified two CaM-binding sites in Cx32: one at NT (res. 15–27, in bold letters) and one at CT1 (res. 209–221, in bold letters) [97], by a program developed by Dr. F.W. De Grado (DuPont the Nemours and Co.):

Res. 1-MNWTGLYTLLSGVN**RHSTAIGRVWLSV**IF**-**29

Res. 208-E**VVYLIIRACARRA**QRRSNPPSRKGSGFGH-238

Nine years later, Török and coworkers, studied the binding of CaM to peptides matching the sequences of the NT and CT1 domains of Cx32 by a fluorescent derivative of CaM (TA-CaM) [98] and by equilibrium fluorescence techniques [76]; both peptides interacted with TA-calmodulin in a Ca^2+^-dependent way [76]. In a later study, the lobe-specific binding of CaM to Cx32 peptides was studied by stopped-flow kinetics on Ca^2+^-binding-deficient mutants of CaM [75]. Peptides that matched the Cx32’s NT domain (MNWTGLYTLLSGVNRHSTAIGR, res. 1–22) bound to both the NH_2_- and the COOH-terminal lobes of CaM (N- and C-lobes), but with higher affinity to the C-lobe. In contrast, the peptides matching the CT1 domain (AEVVYLIIRACARRAQRRSNP res. 208–227) bound to either CaM lobe, one lobe at a time [75].

**Figure 5:**
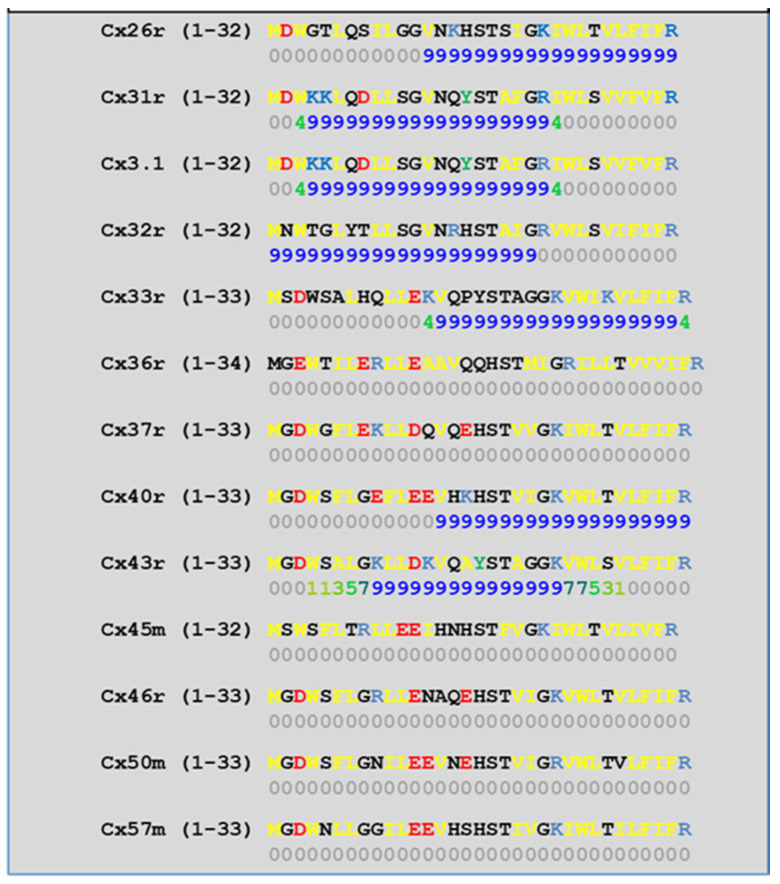
Predicted CaM-binding Site at NT domain.

**Figure 6:**
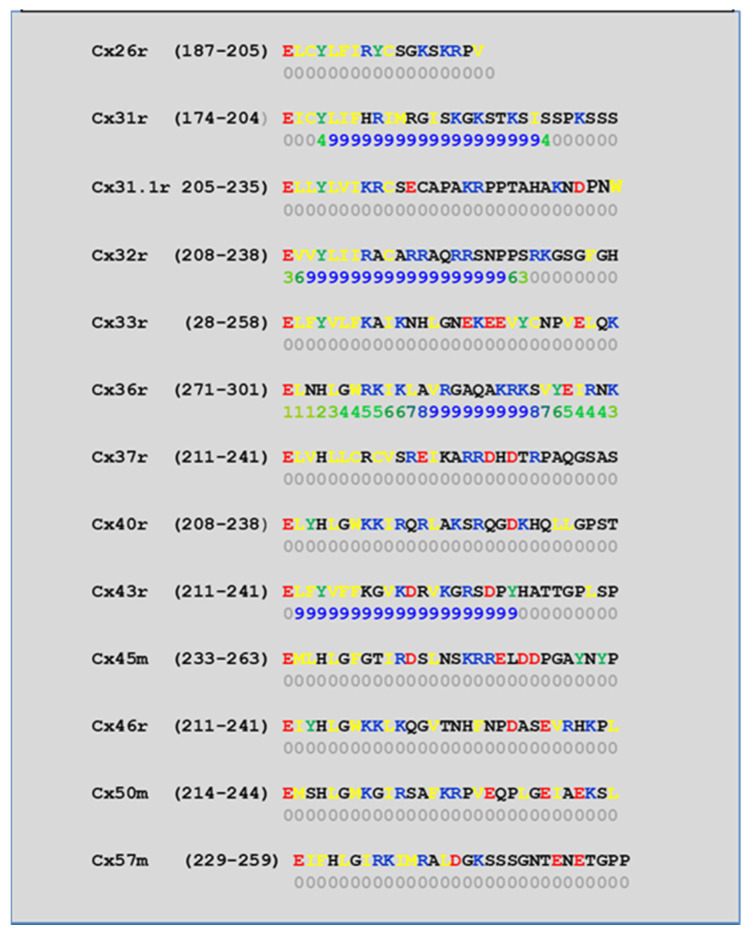
Predicted CaM-binding Site at CT1 Domain.

We have studied the CaM-binding prediction to the NT and CT1 domains of thirteen murine connexins by means of a computer program developed at the University of Toronto (http://calcium.uhnres.utoronto.ca/ctdb/ctdb/sequence.html, accessed on 28 November 2021. Copyright © 2021 Ikura Lab, Ontario Cancer Institute. All Rights Reserved). Figure 5 and Figure 6 show that seven connexins (Cx26, 31, 31.1, 32, 33, 40, and 43) have a potential CaM binding site at NT and only four connexins (Cx31, 32, 36, and 43) have a potential CaM site at CT1.

The CaM binding to the CT1 of Cx32 was confirmed by measuring the interaction with Isothermal Titration Calorimetry (ITC) and Nuclear Magnetic Resonance (NMR) [99]. In this work, which used a longer chain of amino acids (res. 217–283), both CaM lobes interacted with the peptide. In a later study, the CT domain of Cx43, res. 264–290, has also been found to bind CaM [100]. While the CT domain of Cx35 and Cx36 has been reported to be relevant for gating [101], those of Cx43 and Cx32 do not appear to be relevant because Cx43’s CT deletion at res. 257 [102] and Cx32’s CT deletion by 84% [61] do not affect gating sensitivity.

CaM was reported to bind to the CT1 of mouse Cx35, Cx36, and Cx34.7 [103,104]. The CaM binding of CaM to the CT1 domain of Cx36 was further studied by Nuclear Magnetic Resonance (NMR); this work demonstrated that CaM interacts with a peptide matching the CT1 domain in a typical compact state to eight mostly hydrophobic residues (res. 277–284) [104]. The complex Cx36-CaM preceded the assembly of the Cx36 into plaques and allowed dye coupling [104]. Most relevant is the evidence that CaM inhibitors or W277 residue mutation, a residue relevant for CaM-Cx36 interaction, prevented dye coupling [104]. Data for the interaction between CaM and Cx36 before plaque formation also confirm the role of CaM in gap junction formation [96]. The relevance of the CT1 domain in the gating of Cx35 channels was recently confirmed by Aseervatham and coworkers [101].

The study of Burr and coworkers [103] revealed that the Ca^2+^-CaM dissociation constants (K_D_’s) of high-affinity sites ranges from 11-72 nM, and K_1/2_’s for the Ca^2+^ ranges from 3–5 µM. A nM Ca^2+^-CaM sensitivity is higher than expected, but it might be consistent with evidence for the insensitivity of Cx36 channels to 100% CO_2_, which lowered the pH_i_ to ~6.5 [105]. A possible reason for it is that cytosolic acidification did not increase [Ca^2+^]_i_ to a level sufficient for channel gating. In these Cx36-expressing cells, channel gating occurred with alkalinization, which may result from a high pH_i_-induced increase in [Ca^2+^]_i_ [106]. Indeed, cytosolic alkalinization of insect cells increased [Ca^2+^]_i_ and uncoupled the cells at pH_i_ > 7.8 [107].

### 4.2. Cytoplasmic Loop (CL) CaM-Binding Sites

**Figure 7:**
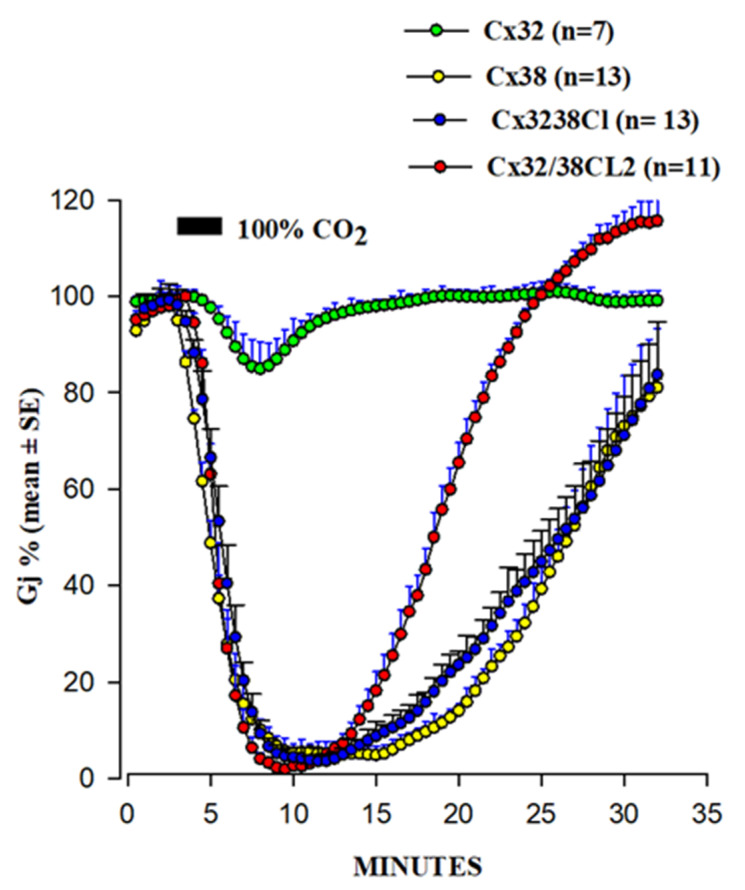
Gj drop induced by 100% CO_2_ in oocyte pairs expressing Cx32, Cx38, or Cx32/38 chimeras. Channels made of Cx32/38CL (Cx32’s CL replaced with that of Cx38) or Cx32/38CL2 (Cx32’s CL2 replaced with that of Cx38) reproduce the gating efficiency of Cx38 channels, although Gj recovers faster in Cx32/38CL2 channels. Adapted from refs. [108,109].

In 1996, we began testing in *Xenopus* oocyte pairs the CO_2_ gating sensitivity of channels made of chimeras and mutants of Cx32 and Cx38, two connexins whose channels are at the opposite end of the spectrum in chemical gating sensitivity [108,109] (Figure 7). Indeed, a 3 min exposure to 100% CO_2_ reduces the Gj of Cx38 channels to zero at a maximum rate of 25%/min, while it reduces that of Cx32 by only ~15% (Figure 7). Even a 15 min exposure to 100% CO_2_ only reduces Cx32’s Gj by ~50%, at the slow rate of 9%/min [108,109].

Channels made of the chimera Cx32/38CL (CL of Cx32 replaced with that of Cx38) matched very well the gating efficiency of the Cx38 channels in the magnitude and in the rate of both the uncoupling and the recoupling (Figure 7). In contrast, the channels expressing the chimera Cx32/38NT (Cx32’s NT replaced by that of Cx38) behaved more like Cx32 than Cx38 channels [109]. Channels made of Cx38 are more Vj-sensitive than those of Cx32 [108,109]. The Cx32/38CL chimera had a Vj sensitivity closer to that of the channel made of Cx38, while the channels made of Cx32/38NT had very low Vj sensitivity [109]. These data point to the important role that CL plays in the sensitivity of both chemical and Vj gating [108].

To identify in more detail the CL domains most relevant to chemical gating sensitivity, we tested Cx32/Cx38 chimeras in which either the first half (CL1) or the second half (CL2) of Cx38’s CL replaced those of Cx32 [108] (Figure 7). Channels made of the chimera Cx32/Cx38CL2 (Cx32 with CL2 of Cx38) matched those made of Cx38 in CO_2_ sensitivity, but the Gj recovered more quickly that in the Cx38 channels, although they matched in the Vj sensitivity of the Cx32 channels (Figure 7). The chimera Cx32/Cx38CL1 (Cx32 with CL1 of Cx38) could not be studied because the functional channels were not expressed. These data indicate that CL1 and CL2 have sequences relevant to fast Vj and chemical gating, respectively [108]. The importance of CL2 in the sensitivity of chemical gating is consistent with the data from recent studies which have identified a CaM binding site in the CL2s of Cx43, Cx50, and Cx44 (rev. in [52]), as well as in Cx32, Cx35, Cx45, and Cx57 [73,74].

**Figure 8:**
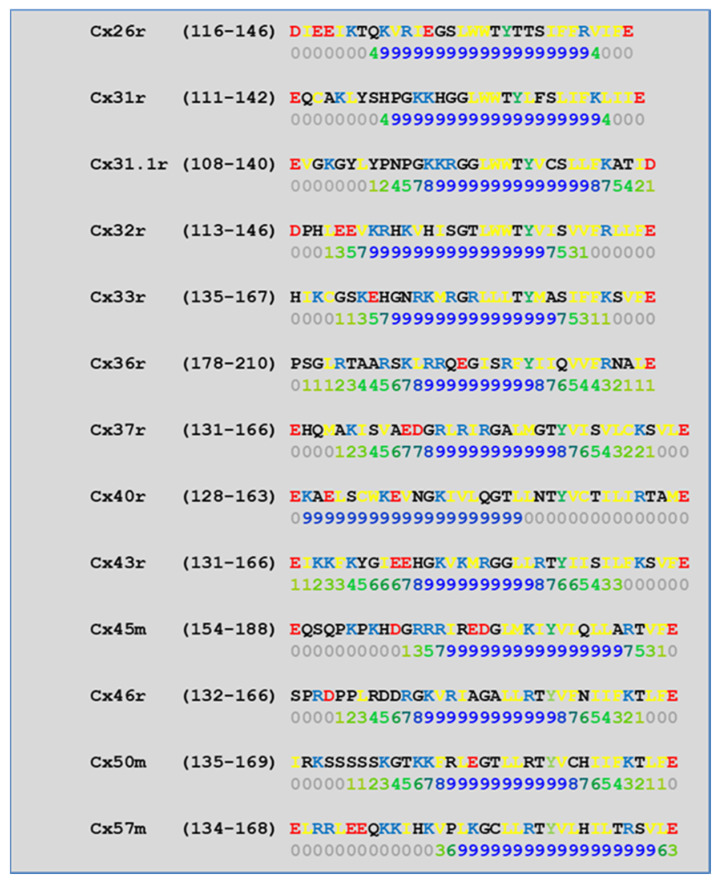
Predicted CaM-binding Site at CL2 domain.

CL2 is likely to be the most relevant CaM binding site in connexins, because in our analysis of CL2 CaM-binding sites (Figure 8) by a computer program developed at the University of Toronto (http://calcium.uhnres.utoronto.ca/ctdb/ctdb/sequence.html, accessed on 28 November 2021. Copyright © 2021 Ikura Lab, Ontario Cancer Institute. All Rights Reserved), all of the thirteen murine connexins tested contain a potential CaM binding site (Figure 8).

Our data indicating that CL2 contains a domain relevant for chemical gating [108] were first confirmed by the evidence for the presence of a CaM binding site in CL2 of Cx43 (res. 136–158) [80]; a peptide matching this domain bound Ca^2+^-CaM with 1:1 stoichiometry, when studied by surface plasmon resonance, circular dichroism, fluorescence spectroscopy, and NMR. In experiments using far-UV circular dichroism, the α-helical content of the peptide increased with the CaM binding. This was further confirmed by fluorescence and NMR studies, which demonstrated that both the CaM and the peptide change in conformation with the peptide-CaM complex formation. The K_D_ of peptide binding to CaM in physiologic potassium concentration ranges from 0.7–1 µM. Upon peptide binding to CaM, the K_D_ of Ca^2+^ for CaM dropped from 2.9 ± 0.1 µM to 1.6 ± 0.1 µM, and the Hill coefficient (*n*_H_) rose from 2.1 ± 0.1 to 3.3 ± 0.5 [80]. For testing the gating competence of Cx43 mutants without the CaM-binding site, two mutants, bound to EYFP (a fluorescent protein), were tested in HeLa cells. Significantly, the absence of the CaM-binding site at CL2 eliminated the Ca^2+^-dependent gating, which was consistent with the idea that the CL2 domain spanning res. 136–158 contains the CaM-binding domain most important for the Ca^2+^-dependent gating of the Cx43 channels [80].

The importance of the CaM-binding site at CL2 was further proven with the Cx43 [42], Cx50 [78], or Cx44 [79] channels; rev. in [52]. A study [110] employed a synthetic peptide that matched the CL2’s CaM-binding site of Cx43 (res. 144–158 KVKMRGGLLRTYIIS) to evaluate by small-angle X-ray scattering of the Ca^2+^-induced changes in the conformation of the CaM-peptide complex. Upon peptide binding, CaM lost the dumbbell shape and became more globular, suggesting that CaM interacts with the peptide in a typical ‘collapsed’ conformation [110].

Xu and coworkers [42] studied whole-cell patch-clamp N2a cells expressing human Cx43 or Cx40. The ionomycin treatment of N2a cells expressing Cx43 tripled [Ca^2+^]_i_ and induced a 95% drop in the Gj; in contrast, in Cx40-expressing N2a cells ionomycin did not significantly change the Gj. The apparent gating incompetence of human Cx40 [42] seems to contradict our evidence for the significant chemical gating sensitivity of the rat Cx40 [111], but it might be consistent with the Ca^2+^-CaM-gating role. Indeed, a computer analysis of CL2’s putative CaM-binding sites shows that the absence of the residues V38 and V43 in rat Cx40, which are replaced by G39 and A44, respectively, in the human Cx40 may be the reason for the predicted inability of the CL2 domain of human Cx40 to bind CaM [50]. With Cx43, the Ca^2+^-induced Gj drop was prevented by CDZ treatment and reversed by the addition of 10 mM EGTA to Ca^2+^-free saline [42]. Addition to the pipette solutions of a Cx43 peptide matching the CL2 CaM-binding domain (res. 136–158) also prevented gating, whereas neither a scrambled peptide nor the Ca^2+^/CaM-dependent kinase II inhibitory peptide (res. 290–309) did [42]. These data confirm that the CaM-binding domain of CL2 is a key player in Cx43’s gating. Table 1 summarizes the potential CaM-binding sites of thirteen murine connexins.

### 4.3. CaM Is Anchored to Connexins at Resting [Ca^2+^]_i_

A number of reports indicate that CaM is anchored to connexins at resting [Ca^2+^]_i_; rev. in [51]. This has recently been confirmed by in vitro experiments that tested the CaM interaction with peptides matching the CL2’s CaM binding site of Cx32, Cx35, Cx45 and Cx57, in the presence and absence of Ca^2+^ [73,74]. In this study, fluorescence changes of the FRET-probe DA-CaM and Ca^2+^-sensitive TA-CaM were recorded by fluorescence spectroscopy and stopped-flow fluorimetry [98] at physiological ionic strength (pH 7.5, 20 °C). Both the Ca^2+^-dependent and the Ca^2+^-independent interactions were found, with the following K_D_ values (Table 2).

FRET measurements demonstrated partial compaction of DA-CaM (54–70% quenching with Ca^2+^ and 33–62% quenching in Ca^2+^-free solutions). The kinetic data showed a two-step process: rapid binding followed by isomerization. This supports the idea that the CaM is anchored to the Cxs and upon stimulation becomes fully bound to them [73,74].

Significantly, the CL2 peptides of Cx45 and Cx57 bind to the CaM with similar high affinities, both with and without Ca^2+^. This seems to suggest that the CaM is anchored by the C-lobe at the resting Ca^2+^, either in a Ca^2+^-free or in a Ca^2+^-bound state. However, one should realize that in vitro peptide-CaM interaction does not say much about the actual channel gating mechanism and the potential conformational changes in connexins that result in channel gating.

## 5. Calmodulin-Cork Gating Model

In 2000, we proposed a CaM-based “cork-type” mechanism of gap junction chemical gating [112]. This “cork” model envisions a physical obstruction of the cytoplasmic mouth of the channel by a CaM lobe [19,50,112,113], probably combined with conformational changes in the connexins, caused by Ca^2+^-CaM binding to the gating site. The model is based on numerous findings that suggest a direct CaM role in gating; rev. in [50,51,52,113]. Experimental evidence indicates that the chemical/slow gate is a sizable, negatively-charged particle, likely to be a CaM lobe [69,114].

**Figure 9:**
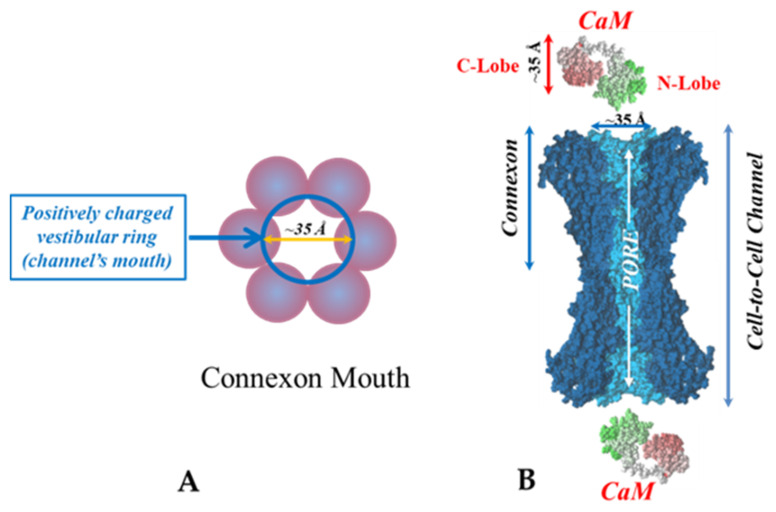
Both the positively charged channel’s mouth (**A**) and the negatively charged CaM lobes (**B**) are ~35 Å in diameter. Therefore, a CaM lobe could fit well in the channel’s mouth (vestibule) and electrostatically interact with it. In B, the channel is split along its length to display the pore diameter (light blue area) along the channel’s length. Both CaM and connexon images (**B**) were provided by Dr. Francesco Zonta (Venetian Institute of Molecular Medicine, VIMM, University of Padua, Italy).

There are many reasons why we think that CaM is the most likely gating candidate. [Ca^2+^]_i_ in the high nM to low μM values activates chemical gating; rev. in [19,52]. In view of the fact that the cytoplasmic domains of connexins do not contain sequences able to bind Ca^2+^ at such low concentrations, the effect of Ca^2+^_i_ on the channel gating is most likely mediated by a CaM-like protein, most likely CaM itself. Indeed, CaM binds to connexins [52,57,58,71,72,77,115] which in fact have CaM-binding sites; most of the sites are at the second half of the cytoplasmic loop (CL2), but some are also at the NH_2_-terminus (NT-site) and at the NH_2_-end of the COOH-terminus (CT1); rev. in [19,52,75,113]. Most relevant for gating are likely to be the CL2 and NT sites [52,73,74,75,77,108,109]. Peptides mimicking the CaM-binding site sequences located at CL2, NT, and CT1 of several connexins bind Ca^2+^-CaM with high affinity [42,52,73,74,75,76,78,79,80,99,103]. Most important is the binding of CaM to the CL2 domain, which has been experimentally confirmed by Jenny Yang’s team for Cx43 [80], Cx44 [79] Cx50 [78], and Cx45 [77] and by Katalin Török’s team for Cx32, Cx35, Cx45, and Cx57 [73,74]. CaM and connexins co-localize at gap junctions (Figure 4) and intracellular sites [71,72,77,104,116]. Recently, the direct binding of CaM to Cx45 has been visualized in living cells by Bioluminescence Resonance Energy Transfer (BRET) [77]; the interaction of CaM and Cx45 was Ca^2+^-dependent and prevented by W7; the CL2’s CaM binding site (res. 164–186) was confirmed by a study reporting its high-affinity interaction (K_D_ = ~5 nM) with a peptide matching the CL2 domain of Cx45’s CL2, tested with a fluorescence-labeled CaM [77]. On the other hand, however, another study provided evidence for both Ca^2+^-dependent and Ca^2+^-independent CaM-binding to the CL2 domains of Cx45, Cx32, Cx35, and Cx57 [73,74]. The Ca^2+^-independent binding of CaM to the CL2 domain [73,74] confirms earlier data suggesting that the CaM is anchored to the Cxs at normal [Ca^2+^]_i_ (~50 nM) [69,71,72,77]. Each of the two negatively-charged CaM lobes is ~25 × 35 Å in size [91], which is the approximate size of the positively-charged cytoplasmic mouth (vestibule) of the channel [117,118,119] (Figure 9). So, a CaM lobe would fit nicely in the mouth (vestibule) of the connexon (Figure 9B). Evidence from a three-dimensional electron density map of isolated gap junctions, which display crystalline (hexagonal) channel arrays (see in the following), studied by X-ray diffraction, proves that the channels are in a closed state as they are inaccessible to sucrose due to a blocking particle at both channel ends similar in size to a CaM lobe [120,121,122] (see in the following). Significantly, in a double-whole-cell-clamp (single-channel) study the chemical/slow gate opens and closes fully and very slowly (transition time = ~10 ms) [123] and the open-to-closed channel transitions, and vice versa, often displayed fluctuations [123]. This further supports the idea that a large particle may transiently flicker in and out of the mouth of the channel before closing the channel completely [123].

We are proposing two types of CaM-mediated cork-gating mechanism: “Ca-CaM-cork” and “CaM-cork”. In the former, the gating involves Ca^2+^-induced CaM activation. In the latter, gating would occur without a [Ca^2+^]_i_ rise above the resting values and in most cases would require either a connexin mutation [69,114] or the application of large Vj gradients [124].

### 5.1. Ca-CaM-Cork Gating Mechanism

**Figure 10:**
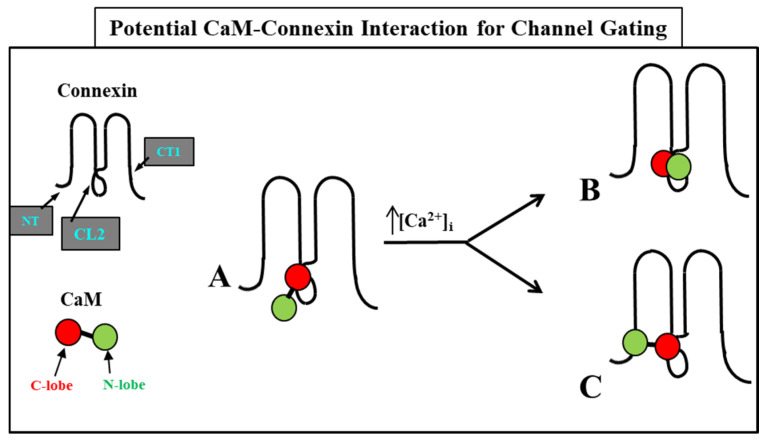
We believe that CaM is anchored to CL2 by its C-lobe lobe (**A**). [Ca^2+^]_i_ > ~50 nM are believed to cause the N-lobe to gate the channel by interacting hydrophobically and electrostatically with CL2 (**B**) or NT (**C**) domains of the same connexin or another connexin of the same connexon (trans-domain or trans-subunit interaction, respectively), resulting in pore blockage (cork gating model).

The Ca-CaM-cork gating envisions that a [Ca^2+^]_i_ rise above resting levels (>~50 nM) causes a CaM lobe (most likely the N-lobe) to block the channel’s mouth (vestibule) by electrostatically and hydrophobically interacting with a receptor site located at CL2 or NT (CaM-gating site) (Figure 10). This interaction is also likely to induce a change in the connexin conformation. The CaM-gating site is likely to be close the channel’s mouth (vestibule) [19,112]. At basal [Ca^2+^]_i_ (~50 nM), the CaM is believed to be anchored to each of the six connexins by one of its lobes (most likely the C-lobe), while the other lobe is likely to be free, but unable to access the channel’s mouth at resting [Ca^2+^]_i_ (Figure 10).

The Ca^2+^-affinity constant of the C-lobe’s EF-hand pair is almost one order of magnitude greater than that of the N-lobe’s EF-hand pair (K_D(app)_ = 5.6 μM and 32 μM for C-lobe and N-lobe, respectively) [125,126]. Therefore, the N-lobe most likely interacts with the channel’s gating site (CL2 or NT) only with an increase in [Ca^2+^]_i_ above the resting values (>~50 nM) (Figure 10).

The Ca-CaM-cork gating is likely to be either reversible or irreversible (Ca-CaM locked gate; see in the following). The former may be activated by a moderate increase in [Ca^2+^]_i_, while the latter may result from a greater increase and/or a more prolonged [Ca^2+^]_i_ rise. We believe that the ultrastructural correlate of the “irreversible Ca-CaM locked gate” is reflected by gap junctions displaying “crystalline” (hexagonal) channel arrays [18,127,128,129,130] (see in the following).

### 5.2. CaM-Cork Gating Mechanism

#### 5.2.1. CaM-Cork Gating in Mutant-Cx32 Channels

The gating behavior of Cx32/mutant heterotypic channels [69,114], Cx45 homotypic channels [70], and Cx32 homotypic channels exposed to large Vj gradients [124] may exemplify the CaM-cork gating mechanism. At the mutant side of the Cx32/mutant channels, the CaM’s N-lobe is believed to engage with the channel’s mouth at basal [Ca^2+^]_i_ (~50 nM) as the mutations might have caused the channel’s mouth to be unprotected, making it accessible to the CaM’s lobe. The CaM lobe would electrostatically interact with the positively charged channel’s mouth so that it could be displaced by Vj positive at the mutant side [69,114]. Indeed, the connexins’ cytoplasmic domain has a high number of basic/acidic residues; in Cx32, for example, if we neglect most of the CT, whose deletion by over 80% does not affect chemical gating [61], one finds 18 basic and 6 acidic residues per connexin if one assigns a value of 1 for R, K, D, and E and a value of ½ for H. In our model, the CaM is anchored to the connexin by its C-lobe at basal [Ca^2+^]_i_, while the N-lobe is free to interact with the channel mouth of the mutant’s hemichannel.

#### 5.2.2. CaM-Cork Gating of Homotypic Cx32 Channels Can Be Activated by Large Vj Pulses

**Figure 11:**
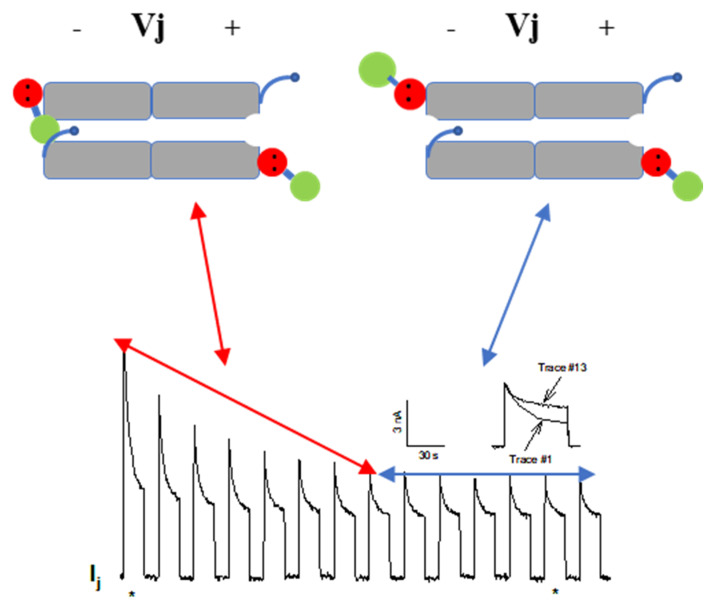
Gj slowly decays in oocytes expressing Cx32 subjected to long −100 mV Vj pulses. Note the progressive drop in Ij-Peak and, to a lesser extent, Ij-Steady-State. Gj peak drops exponentially by 50–60%, eventually reaching steady state. The large drop in Ij during the first 8 pulses is likely to result from the closure of both chemical/slow gate and fast Vj gate (double-headed red arrow). The Ij behavior of the following 6 Vj pulses is likely to reflect the closure of only the fast Vj gate of the remaining open channels (double-headed blue arrow). The CaM molecules are labelled in red and green colors to identify C-lobe and N-lobe, respectively. The inset shows a comparison of the time course of I_j_ seen in traces #1 and #13 (the asterisks below the main tracing indicate their location). Adapted from ref. [124].

The effect of large Vj gradients on the Gj in homotypic Cx32 channels [124], suggests that the gating particle (CaM’s N-lobe) can plug the mouth (vestibule) of the hemichannel at the negative Vj side even in the absence of Cx mutations or [Ca^2+^]_i_ rise (Figure 11). This would suggest that the hemichannel mouth is not totally inaccessible to the CaM’s N-lobe, such that large Vj gradients (100 mV) at the negative side of Vj can force the N-lobe to access the mouth of the channel. Incidentally, these data also confirm earlier evidence for the Vj sensitivity of the chemical gate (the negatively charged CaM’s N-lobe) [69,114].

#### 5.2.3. CaM-Cork Gating in Homotypic Cx45 Channels

In some connexins, large Vj gradients may not be needed to force the gating element (CaM’s N-lobe) into the channel’s mouth at resting [Ca^2+^]_i_. This may be the case in the Cx45 channels as many Cx45 channels are thought to be closed by the chemical/slow gate even at Vj = 0 and without treatments with chemical uncouplers [70,131]. Perhaps, in contrast to the Cx32 channels, in the Cx45 channels the positively-charged ring of the channel’s mouth is conformed in a way that it is spontaneously accessible to the gating element (CaM’s N-lobe) even in the absence of Vj.

#### 5.2.4. How Many CaM’s Lobes Are Needed to Close a Channel?

**Figure 12:**
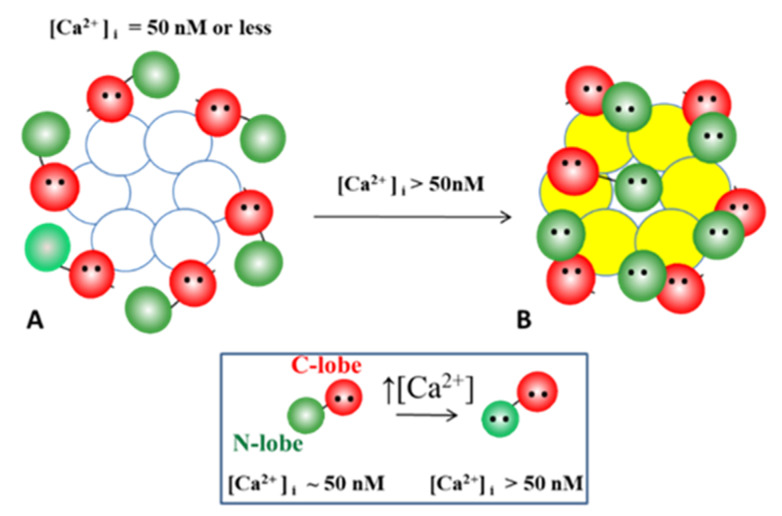
On the basis of the Ca-CaM-Cork model, at normal [Ca^2+^]_i_ (~50 nM) CaM is linked by the C-lobe to each connexin forming a hemichannel (**A**). With a [Ca^2+^]_i_ rise, the N-lobe plugs the pore. Although all of the six CaM N-lobes could theoretically simultaneously bind to their receptor and gate the channel, this seems unlikely because of steric hindrance. It is more likely that just one of the six N-lobes binds to its receptor site (**B**). If so, the first Ca^2+^-activated CaM-lobe wins the competition (first come, first served) and prevents other N-lobes from accessing the mouth of the channel.

With a [Ca^2+^]_i_ rise sufficient to activate the N-lobe of the CaM, how many N-lobes are expected to participate in the channel gating? If indeed the CaM is anchored to each of the six connexins of a connexon, could all of the six N-lobes simultaneously interact with their receptor for gating the channel? This might be possible, but we think it is unlikely due to steric hindrance and because the negatively charged CaM’s N-lobes would electrostatically repel each other. More reasonable, in contrast, is the possibility that channel gating is caused by the interaction of just one N-lobe with the receptor site, either in the same connexin (trans-domain interaction) or in another connexin (trans-subunit interaction) of the connexon (Figure 12).

It is likely that when [Ca^2+^]_i_ rises near the connexon, the first N-lobe that is activated accesses the channel’s mouth, preventing the access of the other N-lobes. Perhaps, the gating N-lobe fluctuates in and out of the channel’s mouth or takes turns with another N-lobe before stably gating the channel. Indeed, while studying, by dual cell clamp, the chemical/slow gating of single Cx43 channels, we reported examples of fluctuating transitions from open to closed states and vice-versa [123]. It is possible that the fluctuating transitions reflect the momentary enter–exit activity of individual N-lobes. This is also consistent with the idea that the gate consists of a relatively large particle (CaM’s N-lobe).

### 5.3. Ca-CaM-Locked-Gate—Irreversible Channel Gating

There is evidence that in some cases the chemical gating of gap junction channels becomes irreversible [42]. Xu and coworkers tested the Ca^2+^-induced uncoupling of N2a cell pairs expressing Cx43 treated with 1 µM ionomycin in 1.8 mM [Ca^2+^]_o_ [42]. The Gj drop caused by the ionomycin treatment was prevented by the calmidazolium or competitive peptides. To test the gating reversibility, at the end of the ionomycin treatment the cells were exposed to a no-Ca^2+^-added solution containing 10 mM EGTA. Significantly, if the switch to the no-Ca^2+^-EGTA occurred when the Gj had only dropped by 50%, the Gj fully recovered, whereas if the switch occurred when the Gj had dropped to 0%, the Gj only recovered to ~60%, suggesting that ~1/2 of the channel population remained irreversibly closed. This indicated that two types of gating state may exist: one reversible and the other irreversible—closed-gate and locked-gate, respectively.

#### 5.3.1. Gap Junction Crystallization and the Locked-Gate Model

**Figure 13:**
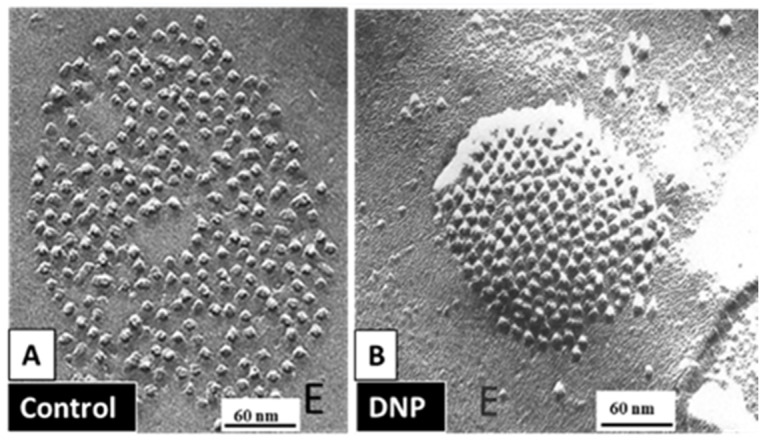
Freeze-fractured gap junctions (E face) of crayfish lateral giant axons fixed in coupled (**A**) or uncoupled (**B**) conditions. Uncoupling was caused by treatment with the metabolic inhibitor dinitrophenol (DNP). With DNP treatment, the channels’ arrays switched from loose (**A**) to tightly (hexagonal) packings (**B**), as the center-to-center spacing between channels decreased from ~200 Å (**A**) to <170 Å (**B**). Adapted from ref. [128].

**Figure 14:**
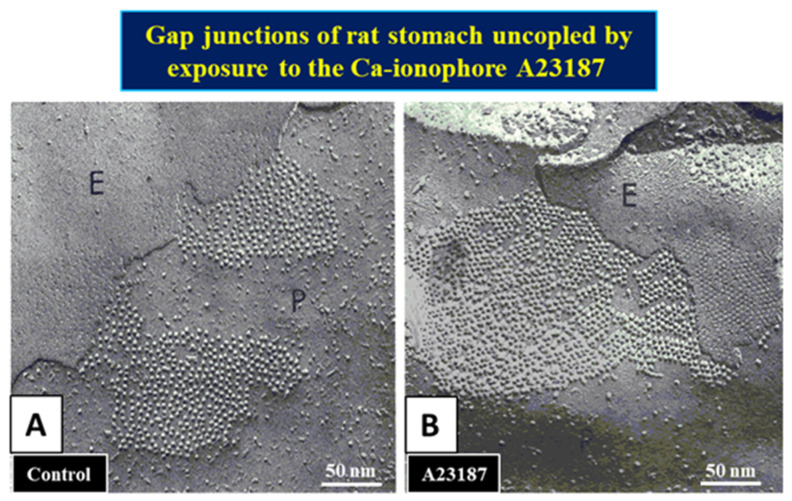
Freeze fractures of rat stomach gap junctions. In samples fixed immediately or kept for 1 h in an oxygenated saline at 37 °C the gap junctions show channels irregularly arranged at center-to-center spacings of 103–105 Å (**A**). In contrast, in samples kept in salines containing 2 µM A23187 (Ca^2+^-ionophore), the channels pack into crystalline, hexagonal arrays with average center-to-center spacings of ~85 Å (**B**). Adapted from ref. [18].

Evidence for an irreversible “locked-gate” state [42] brought to mind our early studies in which were reported changes in gap junction channel aggregation from loose to tight crystalline (hexagonal) arrays, both in invertebrate (Figure 13) and in mammalian (Figure 14) cells, subjected to treatments that uncoupled the cells by increasing [Ca^2+^]_i_ [17,18,62,127,128,129,130,132]. We interpreted these data to reflect a switch from an open- to a closed-channel state.

**Figure 15:**
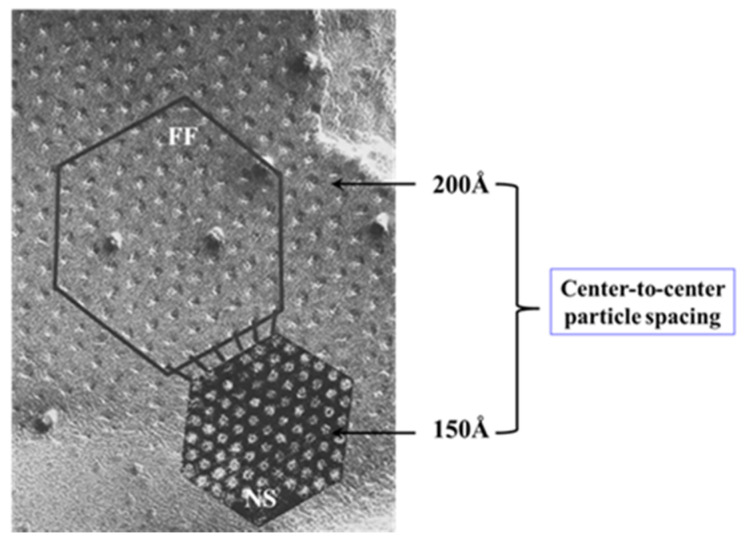
Gap junctions of crayfish lateral giant axons seen in freeze fracture (FF) and in negatively stained isolated junction fragment (NS). In gap junctions fixed in coupled conditions the channels are packed in loose arrays at ~200 Å center-to-center spacing (FF). In contrast, in isolated, negatively stained samples the channels are tightly packed in tight crystalline, hexagonal arrays at 150–170 Å center-to-center spacing (NS), as in axons exposed to treatments known to increase [Ca^2+^]_i_ (see Figure 13B). We interpreted these changes to reflect a switch from open- to closed-channel state. We believe that gap junction crystallization represents an irreversible “locked-gate” gating state. Adapted from ref. [128].

**Figure 16:**
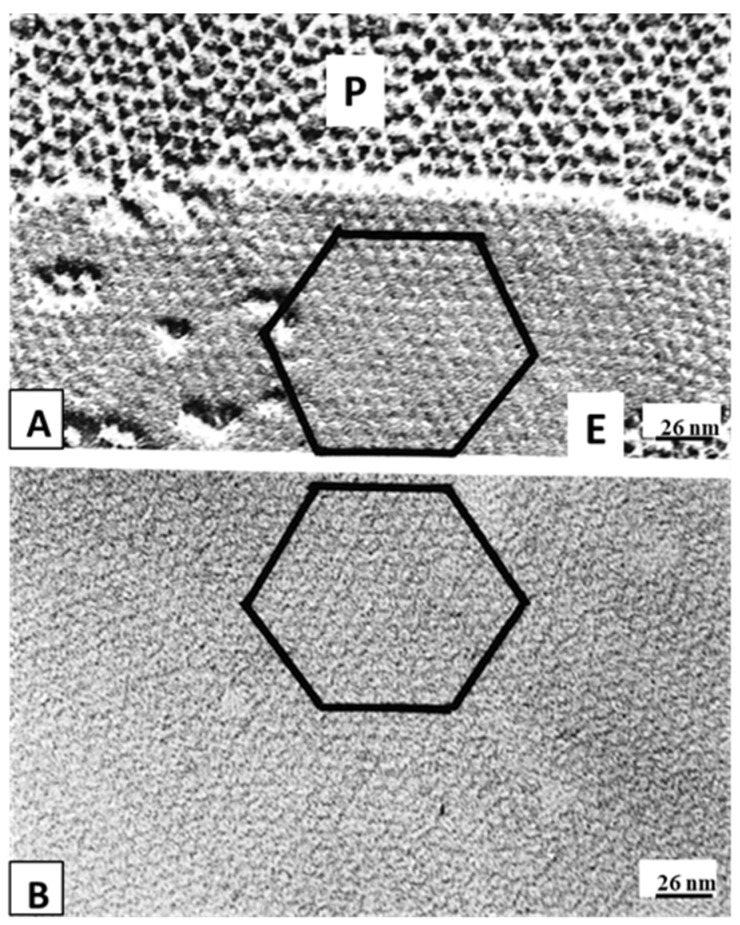
Gap junctions of rat liver epithelium (in uncoupled state) from a freeze-fracture replica (**A**) and an isolated, negatively stained junction (**B**). Isolated junctions (**B**) display crystalline (hexagonal) arrays identical to those of uncoupled junctions (**A**). The channels of both gap junctions are believed to be in irreversibly closed state (Ca-CaM locked state). In both junctions, the channels are hexagonally packed at an average center-to-center spacing of −8.5 nm. Adapted from ref. [129].

This interpretation was based on our earlier observation that gap junctions isolated from crayfish nerve cords and stained negatively with phosphotungstic acid (PTA) display tightly packed (hexagonal) arrays with ~150 Å center-to-center spacings [130] (Figure 15), which are the same as those of the gap junctions of axons fixed in an uncoupled state [128] (Figure 15). Crystalline (hexagonal) arrays were also observed in negatively stained gap junctions isolated from rat liver epithelia (Figure 16) [11]. Thus, we proposed that the channels of the isolated gap junction were in a closed state. We felt that gap junction crystallization is likely to reflect an irreversible state of channel gating (locked-gate state). In support of the gated state of isolated gap junctions in the crystalline state is crystallographic evidence demonstrating that the channels are blocked at both ends by a particle [120,121,122], which, significantly, is very similar in size to a CaM lobe (see in the following).

#### 5.3.2. What Causes Gap Junction Crystallization?

**Figure 17:**
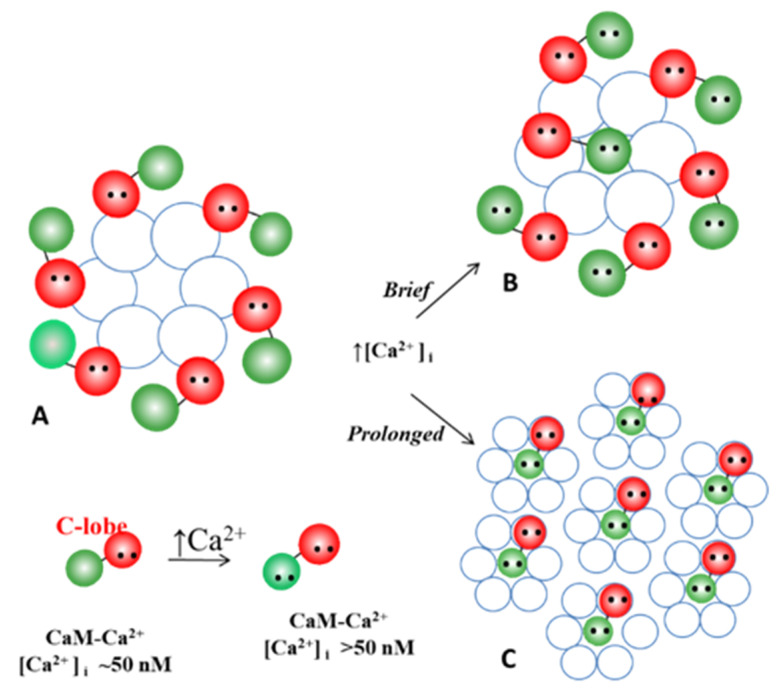
We are proposing that with exposure to high [Ca^2+^]_i_, non-gating CaM molecules, bound to connexins by one lobe (**B**), detach from connexins (**C**), enabling the channels to become tightly arranged into crystalline arrays (**C**), interacting mostly hydrophobically with each other. (**A**) Permeable channel. (**B**) Channel reversibly gated. (**C**) Irreversibly closed channel (“lock-gated”).

Our hypothesis proposes that that the loose, less ordered particle array of coupled junctions is caused by the presence of CaM molecules linked to each of the six connexin of the hemichannel (Figure 17A). We think that in coupled junctions a CaM molecule is bound to each connexin by the C- lobe, while the N-lobe is free, such that there would be six unbound CaM N-lobes per hemichannel (Figure 17A). Due to the negative charge of the CaM lobes, the N-lobes would be repelling each other, such that the channels would be separated (Figure 13A and Figure 14A). In coupled junctions, the channels would be spaced at a ~100 Å center-to-center distance in vertebrates and a ~200 Å in crayfish axons, and the junctions would display loose and irregular channel arrays. Based on the Ca-CaM-cork model, moderate [Ca^2+^]_i_ rise would cause a CaM’s N-lobe to reversibly close the channel (Figure 17B). This type of “reversible” gating state (Ca-CaM-cork) would not cause gap junction crystallization, as the electrostatic repulsion among the other five N-lobes would prevent it. So, what is causing gap junction crystallization?

Our idea is that with prolonged exposure to high [Ca^2+^]_i_ all but one CaM molecule may detach from the connexins (Figure 17C). This would eliminate the repulsive force among channels, causing the channels to tightly aggregate into crystalline (hexagonal) arrays (Figure 13, Figure 14, Figure 15 and Figure 16 and Figure 17C) and interact with each other mostly hydrophobically. This could be the reason why during the process of gap junction isolation the channels remain tightly linked to each other (Figure 15 and Figure 16B).

If this were the case, what would cause the release of CaM molecules from gap junctions at high [Ca^2+^]_i_? Black and coworkers [133] invented a fluorescent biosensor designed for determining [CaM]_i_ of both Ca^2+^-free (apo-CaM) and Ca^2+^-activated (holo-CaM). In a human kidney cell line, they found that the [CaM]_i_ at basal [Ca^2+^]_i_ is 8.8 ± 2.2 µM, but a [Ca^2+^]_i_ increase dramatically decreases the [CaM]_i_ to ≤200 nM because of the extensive buffering of the free CaM by the large increase in the available CaM-binding sites. The same phenomenon with [Ca^2+^]_I_ elevation was observed in rabbit cardiac-myocytes, as the CaM reversibly migrated into the nucleus [134]; this study also reported that with a [Ca^2+^]_i_ rise, the CaM migrates from the Z-line to the Ca^2+^-CaM-binding sites with higher affinity. The same phenomenon may to occur in gap junctions, a possibility being that with a great increase in [Ca^2+^]_i_ the non-gating CaM molecules, linked to the connexins by just one lobe, are released from the connexins.

We are aware that the mechanism for gap junction crystallization we are proposing is highly speculative and needs to be experimentally tested. Perhaps, future work could test this idea by attempting to detect biochemically the presence of CaM molecule in isolated gap junctions. However, one should realize that if only two CaM molecules are bound to each channel of crystalline gap junction fragments, the CaM will only represent 14.3% of the proteins, as each channel is made of twelve connexin monomers.

## 6. The Calmodulin-Cork Model Is Supported by X-ray Diffraction Images of Isolated Gap Junction Channels in Closed State

**Figure 18:**
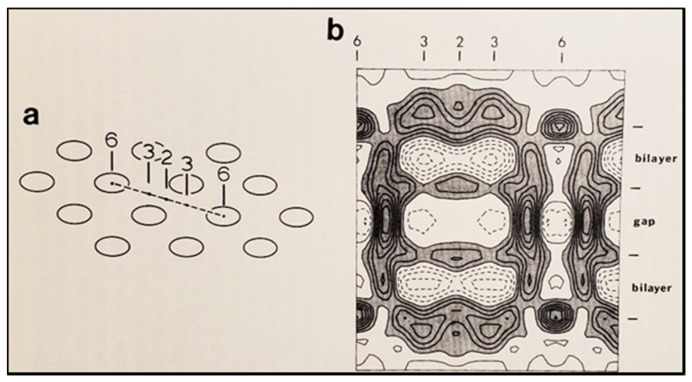
Diagram of the three-dimensional structure of a frozen-hydrated gap junction isolated from mouse liver and solved to 18 Å resolution by X-ray diffraction. The channel “6” in (**a**,**b**) is impermeable to 50% sucrose, proving that it is closed at each cytoplasmic end by a particle that prevents sucrose entry. This proves that the channels of isolated (crystalline) junctions are in closed state (Ca-CaM locked state). Reproduced from ref. [121] with permission from the Journal of Molecular Biology and the Cold Spring Harbor Laboratories.

**Figure 19:**
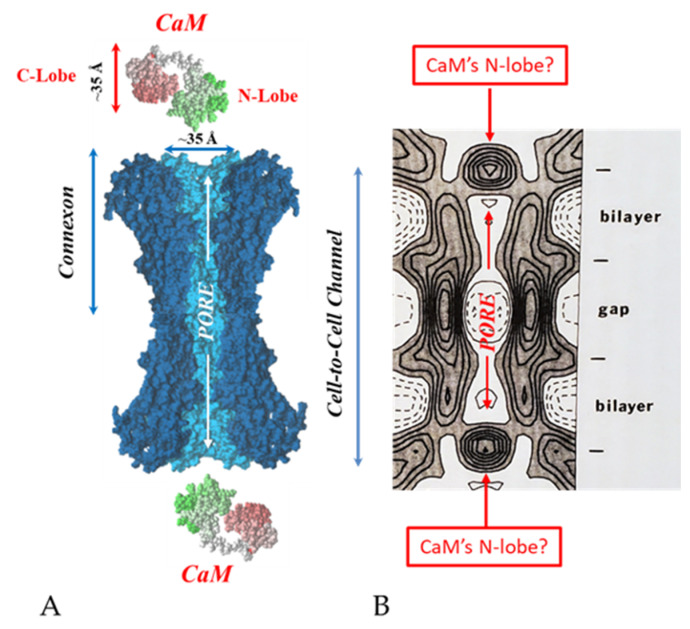
Both the positively charged channel’s mouth and the negatively charged CaM lobes are ~35 Å in size (**A**). Thus, a CaM lobe could fit well within the positively charged connexon’s mouth (**A**). Significantly, the three-dimensional structure of gap junctions isolated from mouse liver (**B**) demonstrates that the channel of isolated (crystalline) junctions is impermeable to 50% sucrose, proving that it is closed at each cytoplasmic end by a particle that prevents sucrose entry (**B**). Our hypothesis is that the blocking particle is the CaM’s N-lobe (**B**). Both the CaM and connexon images (**A**) were provided by Dr. Francesco Zonta (VIMM, University of Padua, Italy. (**B**) was reproduced from ref. [121] with permission from the Journal of Molecular Biology and the Cold Spring Harbor Laboratories.

In the early 1980s, Makowski and coworkers described the structure of crystalline (hexagonal) gap junctions isolated from mouse liver in a high-resistance configuration (closed channels) by analyzing X-ray diffraction data at 18 Å resolution (Figure 18 and Figure 19B) [120,121]. The channels’ gated condition of these isolated gap junctions was proven by the evidence that the channels were impermeable to sucrose [120]; in their words: “Analysis of diffraction patterns from isolated gap junctions in 50% sucrose shows that the sucrose fills the extracellular gap but fails to enter the channel. It is possible that the channel is closed at both cytoplasmic surfaces, excluding sucrose. This suggests that the isolated junctions are in a high resistance state” [120]. Indeed, the three-dimensional map of the electron density demonstrated that the channels were blocked at both cytoplasmic ends by a small particle; in their words: “Its position blocking the channel suggests that it may comprise a gating structure responsible for the control of channel permeability, X-ray diffraction studies of junctions in varying concentrations of sucrose (Makowski et al. 1984a) [122] indicated that in these preparations the channel was closed to the penetration of sucrose and that a solvent region approximately 100 Å long and centered on the six fold axis remained free of sucrose” [121].

Significantly, the blocking particle, spherical in shape, is approximately 30–35 Ǻ in diameter [121] (Figure 18 and Figure 19B), which is remarkably similar in size to a CaM lobe (Figure 19A). Indeed, in their words: “The channel has a diameter of 20–30 Ǻ along most of its length but appears to narrow to a minimum diameter of about 15 Ǻ in the extracellular half of the bilayer. Both the sucrose results and the three-dimensional map are consistent with the idea that a structure located near the cytoplasmic surface of the membraned is blocking the channel in these preparations” [121]. These studies support our evidence that gap junctions with crystalline (hexagonal) channel arrays are in an uncoupled (gated) state (Ca-CaM locked gate, Figure 13, Figure 14, Figure 15 and Figure 16) [11,17,18,127,128,129,130] and suggest that a CaM lobe is gating the channel.

## 7. Conclusions and Future Perspectives

This article has reviewed four decades of data supporting the direct role of CaM in gap junction channel gating. The Ca-CaM-cork gating mechanism [113], proposed over two decades ago [112], is based on evidence from the effect of CaM inhibitors, the inhibition of CaM expression, the expression of a CaM mutant (CaMCC) with higher Ca^2+^-sensitivity, CaM-connexin co-localization at gap junctions, the presence of high-affinity CaM-binding sites in connexins, the expression of connexin mutants, the gating effect of repeated large Vj pulses, data at the single channel level, the recovery of lost gating competency by addition of Ca-CaM to internally perfused crayfish axons and, finally, X-ray diffraction data on isolated gap junction fragments.

One may ask: why is it important to understand in detail the gating mechanism of gap junction channels? It is important not only because cell–cell uncoupling is more than just a safety mechanism for protecting healthy cells from damaged neighbors (healing over), but because the gating sensitivity to [Ca^2+^]_i_ in the high nanomolar range indicates that the fine modulation of direct cell–cell communication provides cells with the means for regulating tissue homeostasis. In addition, and more importantly, the field should be encouraged to test the effect of recently discovered disease-causing CaM mutants on gap junction function [135].

Indeed, recent evidence of diseases caused by CaM mutations [136,137,138,139,140,141] suggests the potential role of CaM mutants in diseases affecting gap junction function. Almost two dozen CaM mutations have been found to cause cardiac malfunctions, most of which occur in the CaM’s C-lobe, one in the N-lobe, and one in the linker between the C- and N-lobes. In most cases the electrocardiogram (ECG) demonstrates the presence of Long QT Syndrome (LQTS), a change that affects the electrical activity of the heart, which is often associated with Catecholaminergic Polymorphic Ventricular Tachycardia (CPVT) phenotype and Idiopathic Ventricular Fibrillation (IVF). CPVT patients manifest ventricular tachycardia that can lead to death by ventricular fibrillation. In most cases, cardiac malfunctions have been attributed to the effect of CaM mutations on the ryanodine receptor (RyR2) and the cardiac L-type voltage-gated Ca^2+^ channel. However, other membrane channels, potential targets of CaM mutants, have also been suggested. Curiously, however, in spite of strong evidence for the direct CaM role in gap junction channel regulation, the potential consequences of these CaM mutants on direct cell–cell communication—a mechanism fundamental for the function of virtually all vertebrate and invertebrate organs—have not yet been addressed. Therefore, it is clear that future efforts should be aimed at testing the effect of these CaM mutants, and the future discovered CaM mutants, on gap junction mediated communication [135].

## Figures and Tables

**Table 1:** Predicted CaM-binding sites in connexins.

Connexin	NT Site	CL2 Site	CT1 Site
Cx26r	Yes	Yes	
Cx31r	Yes	Yes	Yes
Cx31.1r	Yes	Yes	
Cx32r	Yes	Yes	Yes
Cx33r	Yes	Yes	
Cx36r		Yes	Yes
Cx37r		Yes	
Cx40r	Yes	Yes	
Cx43r	Yes	Yes	Yes
Cx45m		Yes	
Cx46r		Yes	
Cx50m		Yes	
Cx57m		Yes	

**Table 2:** CaM binding to CL2.

	K_D_ (with Ca^2+^)	K_D_ (Ca^2+^-Free)
Cx32	40 ± 4 nM	280 ± 10 nM
Cx35	31 ± 2 nM	2.67 ± 0.09 µM
Cx45	75 ± 4 nM	78 ± 1 nM
Cx57	60 ± 6 nM	52 ± 14 nM
